# *Coxiella burnetii* Infection in Roe Deer during Q Fever Epidemic, the Netherlands

**DOI:** 10.3201/eid1712.110580

**Published:** 2011-12

**Authors:** Jolianne M. Rijks, Hendrik I.J. Roest, Peter W. van Tulden, Marja J.L. Kik, Jooske IJzer, Andrea Gröne

**Affiliations:** Dutch Wildlife Health Centre, Utrecht, the Netherlands (J. M. Rijks, M.J.L. Kik, J. IJzer, A. Gröne);; Central Veterinary Institute, part of Wageningen University and Research Center, Lelystad, the Netherlands (H.I.J. Roest, P.W. van Tulden).

**Keywords:** Coxiella burnetii, Q fever, Capreolus capreolus, roe deer, PCR, IS1111a, genotyping, zoonoses, the Netherlands, bacteria, vector-borne infections

**To the Editor:** A Q fever epidemic among humans started in the Netherlands in 2007 and peaked in 2009 (*1*). Epidemiologic evidence linked the epidemic to abortions and deliveries among *Coxiella burnetii*–infected dairy goats and dairy sheep (*1**,**2*). However, questions arose about whether *C. burnetii* infection in free-living wildlife might be another source of Q fever in humans. *C. burnetii* has a wide host range (*3*), but to our knowledge no studies had addressed its occurrence in nondomestic animals in the Netherlands (*4*).

The main objective of this study was to look for evidence of *C. burnetii* infection in carcasses of free-living roe deer (*Capreolus capreolus*) in the Netherlands, where *C. capreolus* is the most common species of wild ruminant. Additional objectives were to 1) analyze characteristics, location, and time of death of case-animals for more information on the infection in roe deer and 2) determine the genotype of *C. burnetii* strains from roe deer and compare them with the genotype of strains from domestic animals and humans for evidence of spillover.

The sample consisted of 79 roe deer that were euthanized or found dead in 9 of the 12 provinces in the Netherlands during January 2008–May 2010. All animals had undergone postmortem examination, and tissue samples were frozen until testing. Tissues tested were lung (n = 46), spleen (n = 50), bone marrow (n = 50), liver (n = 74), and kidney (n = 75), as available. We extracted DNA by using the DNeasy Blood and Tissue Kit (QIAGEN, Hilden, Germany). A duplex quantitative PCR targeting the IS*1111a* element was used with an internal control gene, as described (*2*). Tissues with cycle threshold (C_t_) values <34 (1/case) were typed by using multilocus variable-number tandem-repeat analyses (MLVA) for 11 loci, as described (*2**,**5*); results were compared with known MLVA typing data from the Netherlands.

Of the 79 roe deer examined, 18 (23%) had positive PCR results for *C. burnetii* DNA in multiple (5/18, 28%) or single (13/18, 72%) tissues. The average C_t_ value was 36.30 (range 32.07–39.47). Among 29 roe deer for which all 5 tissues were tested, no single tissue was more frequently positive than others for *C. burnetii* (χ^2^ = 1.07, df = 4, p = 0.9) or had lower C_t_ values (single factor analysis of variance, p = 0.58). These findings indicate that testing multiple tissues per individual enhances case detection.

No specific sex, age, or health effects were observed. Of 48 male deer, 10 (21%) had positive results, compared with 8 (27%) of 30 female deer (1 missing value; χ^2^ = 0.35, df = 1, p = 0.55). Of 50 deer >1 year of age, 15 (30%) had positive results, compared with 2 (15%) of 13 deer <1 year of age (16 missing values; 2-tailed Fisher exact test, p = 0.49). Postmortem findings varied for *C. burnetii*–positive deer.

*C. burnetii* cases occurred in most provinces studied (6/9, 66%) and in all 3 study years. Significantly more *C. burnetii*–positive deer were observed in 2010 (13/30, 43%) than in 2008 (2/18, 11%) and 2009 (3/31, 10%) (χ^2^ = 11.62, df = 2, p < 0.01). This finding might represent sample bias or indicate spatial or temporal clustering in 2010.

The *C. burnetii* genetic material found in roe deer may indicate past or ongoing infection (*6*). Although positive cases occurred in all seasons, those more likely to represent ongoing infection (multiple infected tissues and C_t_ values <36; n = 4) occurred in March, April, and June. Clinical Q fever in roe deer might occur more frequently in late gestation and around parturition, as in domestic ruminants (*7**,**8*). Furthermore, Q fever in wildlife might have its own sylvatic cycle (*4**,**9*). However, analogous to human cases in 2007–2010 (*1*), the pattern could also include spillover events from domestic livestock.

Tissues of 2 springtime case-animals had C_t_ values <34. MVLA typing of these strains yielded partial genotypes (Figure). Comparison with those of strains from domestic dairy animals or humans during 2007–2010 showed that these 2 strains from roe deer differed from the main goat- and sheep-derived strain involved in the Q fever epidemic (genotype CbNL01 [*2*]) and from other strains found (inconclusive for CbNL08; Figure).

**Figure:**
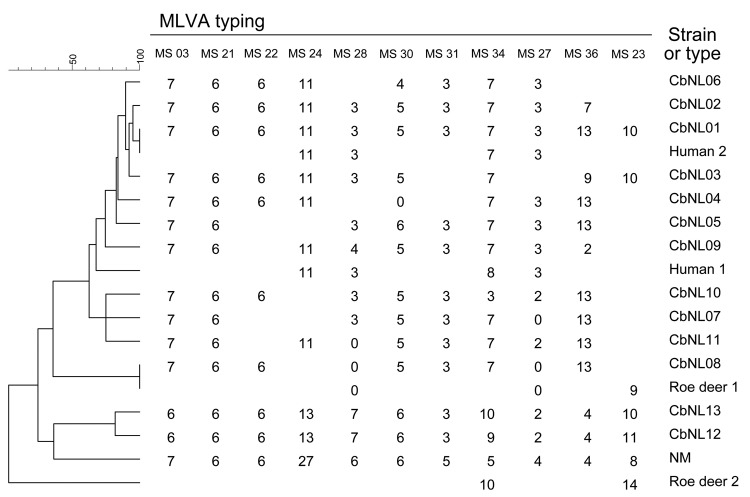
Phylogenetic tree with genotypes of *Coxiella burnetii* from goat, human, and roe deer samples from the Netherlands. Genotypes were determined on the basis of 11 multilocus variable-number tandem-repeat analyses (MLVA). The number of repeats per locus is shown; open spots indicate missing values. Roe deer 1 was an adult female found dead on March 30, 2010, in Friesland Province. Roe deer 2 was a young female deer involved in a traffic accident on April 6, 2010, in Utrecht Province. The goat and human samples have been described (*2*). Scale bar indicates genetic relatedness. Human 1, QKP 1; Human 2, QKP 2; NM, Nine Mile reference strain; MS, MiniSatellite.

Our study confirmed that *C. burnetii* infection occurs in free-living roe deer in the Netherlands. *C. burnetii* DNA was detected in roe deer of both sexes and age groups with no particular health effect, and it was detected in animals in different provinces and in all years studied; the highest *C. burnetii* DNA loads occurred in spring and early summer. Detection of genetic material by PCR does not always imply viable infective bacteria (*6*). However, because the infectious dose of *C. burnetii* is low (*10*), our findings support the use of preventive hygiene measures (*4*) to minimize zoonotic risk when handling roe deer. The 2 MLVA-typed strains provided no evidence for spillover of the predominant strain involved in the Q fever epidemic in the Netherlands. More studies are required to adequately understand Q fever cycles in wildlife and their relationship with Q fever in domestic animals and humans.
